# Implications of the COVID-19 Lockdown on Dengue Transmission in Malaysia

**DOI:** 10.3390/idr13010016

**Published:** 2021-02-05

**Authors:** Song-Quan Ong, Hamdan Ahmad, Ahmad Mohiddin Mohd Ngesom

**Affiliations:** 1 CPUS, UOW Malaysia KDU Penang University College, 32, Jalan Anson, George Town 10400, Malaysia; 2 School of Computer Sciences, Universiti Sains Malaysia, Gelugor 11800, Malaysia; 3 Vector Control Research Unit, School of Biological Sciences, Universiti Sains Malaysia, Gelugor 11800, Malaysia; hamdana@usm.my; 4 Faculty of Health Science, Universiti Kebangsaan Malaysia, Jalan Raja Muda Abdul Aziz, Kuala Lumpur 50300, Malaysia; aksmohiddin@yahoo.com

**Keywords:** *Aedes aegypti*, *Aedes albopictus*, dengue fever, movement control order

## Abstract

We aim to investigate the effect of large-scale human movement restrictions during the COVID-19 lockdown on both the dengue transmission and vector occurrences. This study compared the weekly dengue incidences during the period of lockdown to the previous years (2015 to 2019) and a Seasonal Autoregressive Integrated Moving Average (SARIMA) model that expected no movement restrictions. We found that the trend of dengue incidence during the first two weeks (stage 1) of lockdown decreased significantly with the incidences lower than the lower confidence level (LCL) of SARIMA. By comparing the magnitude of the gradient of decrease, the trend is 319% steeper than the trend observed in previous years and 650% steeper than the simulated model, indicating that the control of population movement did reduce dengue transmission. However, starting from stage 2 of lockdown, the dengue incidences demonstrated an elevation and earlier rebound by four weeks and grew with an exponential pattern. We revealed that *Aedes albopictus* is the predominant species and demonstrated a strong correlation with the locally reported dengue incidences, and therefore we proposed the possible diffusive effect of the vector that led to a higher acceleration of incidence rate.

## 1. Introduction

Dengue fever is a vector-borne disease that threatens over one-third of the world’s population and causes 100 million dengue infections worldwide every year [1]. The disease is always a major health concern in Malaysia, with the incidences having a dramatic increase from 2017 (82,851 incidences) to 2019 (130,004 incidences), which recorded the highest dengue incidences. The dengue fever endemic pattern in Malaysia is seasonal, with variable transmission and prevalence patterns affected by the large diversity in rainfall and spatial variation [2]. Two major transmission periods of dengue fever occur from June to September, following Southwest monsoon rains seasons, and from September to March, following Northeast Monsoon rains seasons that bring higher precipitation and lead to the greater potential breeding ground for the vector [3]. On 18 March 2020, Malaysia had implemented a lockdown to halt COVID-19 transmission, and the duration of lockdown has coincided with the end of Northeast monsoon seasons and the start of the dry season (March to June). Therefore, the impact of human movement restriction during lockdown on dengue transmission has generated considerable research interest. Lockdown has been commonly implemented all over the world to halt COVID-19 transmission [4]. This is to control human movement, as physical proximity of host is a key risk factor for the transmission of COVID-19 [5]. Although the control of human physical proximity is able to shape the spatial spread of a pathogen that is directly transmitted, especially for COVID-19 [6], the effect on indirectly transmitted infectious diseases such as dengue fever remains unclear. Despite dengue fever being the most prevalent mosquito-borne disease in the world [1], studies on the impacts of human movement on the transmission are relatively uncommon; even this information may assist in the prevention system. Most studies have used modelling to simulate the condition of large scale human mobility on dengue transmission, such as Falcón-Lezama et al. [7], who used a mathematical model to evaluate the effect of people’s day-to-day movement on the dengue epidemic and concluded that the vector-host’s spatial connectivity posed an epidemic risk. Stoddard et al. [8] used a model together with the contact-site cluster investigations in a case-control design to review the risk of dengue infection by human movement and argued the importance of movement restriction in managing the spatiotemporal dynamics of the dengue virus. However, the previous experimental configurations were far from the real situation, especially when the mobility of the population on a large scale is not feasible to demonstrate the direct effect on dengue transmission. With the imposition of the COVID-19 lockdown in Malaysia from 12th to 23th weeks of 2020 (18 March to 9 June 2020), about 90% of people were restricted to their homes, and 10% of essential workers were allowed to carry out their daily activities for the whole country [9]; thus, the unprecedented large-scale movement restriction of hosts provides opportunities to evaluate its direct impact on dengue transmission. 

To understand the direct impact of the large-scale movement restriction on the dengue endemic in Malaysia, we compared the actual and previous dengue incidences trend, which is a method that is commonly conducted by previous studies. In order to further investigate the heterogeneity of the trend due to the lockdown, constructing a simulation model that expects no interference could be one of the approaches, as demonstrated by other studies on infectious diseases [10,11]. To construct a best-fitting simulation, due to the dengue endemic in Malaysia being seasonal, a seasonal time series analysis such as Seasonal Autoregressive Integrated Moving Average (SARIMA) is a suitable approach to construct a model, as it is the method always used to predict infection incidences that are seasonal [10,11].

One of the key elements in dengue transmission is the abundance and distribution of the vectors—*Aedes aegypti* and *Aedes albopictus*—in which the mosquitoes’ behavior such as the host-seeking process, is also potentially affected by movement restrictions of humans. By using an agent-based transmission model, Reiner et al. [12] indicated that the socially structured movement of humans caused a significant influence on dengue transmission, and the infection dynamics were hidden by the diffusive effect of the vectors. The implementation of lockdown in Malaysia changed the host’s daily activities, most of the time people were contained in their own housing areas, and also to both *Ae. aegypti* and *Ae. albopictus* that are highly anthropophilic, in which *Ae. aegypti* almost exclusively rely on human blood and *Ae. albopictus* is an aggressive and highly adaptive species that can easily colonize the habitat of other mosquitoes in urban areas [13]. In addition, the spatial distribution of the host also influenced the behavior of the vectors, and previous studies [14,15,16,17,18] identified a shift of the *Ae. albopictus* habitat to an indoor environment where the species usually inhabit the forest or are mostly vegetative and cause interspecies competition with other existing mosquito species, such as *Ae. aegypti*. Therefore, when the COVID-19 lockdown restricts humans in mostly indoor environments with minimum outdoor activities, we are interested in the distribution of the mosquitoes as well.

We study the effect of the physical proximity restriction on humans during the COVID-19 lockdown in Malaysia with two objectives:

Dengue Transmission. This study aims to understand the impact of movement restrictions on the dengue transmission by comparing the trend of dengue incidences of the year 2020 with previous years of incidences, and compared statistically with the confidence level of a SARIMA model. We establish the SARIMA model that expects no human movement restrictions practice from the dataset of Malaysia weekly reported dengue incidences of previous years (2015 to 2019). We evaluate the level of heterogeneity of the actual trends from previous years and simulated a model according to the phases of lockdown that were implemented by the Malaysia Government. To standardize the time-series deception for this study, the phases in the reminder of this articles are stated as “stage” and are accompanied with the corresponding weeks that are detailed in Table 1.

Vectors Occurrences. We aimed to obtain data on the abundance and distribution of *Aedes* mosquitoes during the period of lockdown by conducting sampling in the indoor and outdoor environments of areas from a district of Penang, Malaysia.

## 2. Materials and Methods

### 2.1. Data Collection

We started the analysis from the data of 2015, which recorded the second-highest dengue incidence after 2019 (the rate of dengue incidence on 2015 and 2019 are 2324/week and 2500/week, respectively) [19]. We retrieved the data from the official press statement and the Dengue Surveillance System developed by the Vector-Borne Disease Section, Ministry of Health (MOH), Malaysia, to monitor dengue transmission using remote sensing (RS)—iDengue [19], under the supervision of Remote Sensing Agency Malaysia. This information is updated on a daily basis and can be accessed by the public in the MOH of Malaysia’s official website. This surveillance system monitors dengue transmission across the country by updating dengue fever cases reported in every hospital and medical institution on a daily basis, and all notified cases were followed up by the relevant health authorities for case verification before being recorded in the registry of the Dengue Surveillance System.

### 2.2. Temporal Analysis of Malaysia Dengue Incidences during Lockdown

**Figure 1:**
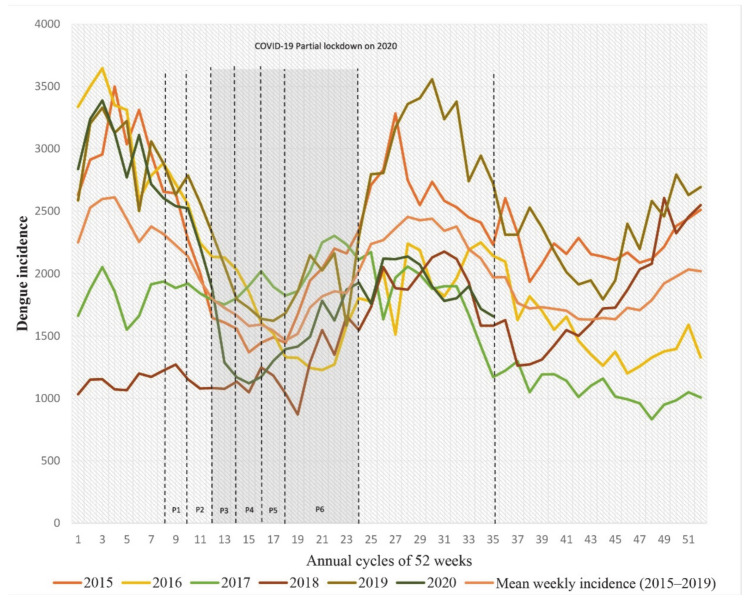
Dengue endemic growth from the years 2015 to 2020. Eight stages: Pre2, Pre–lockdown 2; Pre1, Pre–lockdown 1; P1, Phase 1; P2, Phase 2; P3, Phase 3; P4, Phase 4; P5, Phase 5; P6, Phase 6.

Due to the different scales of movement restriction imposed by the Malaysia government in the year of 2020, we specify that the time period of the Malaysia major lockdown is the 12th week (18 March) to the 23rd week (9 June) of 2020 (Table 1), which involved major border closedown and more than 90% population movement restrictions nationwide [9]; this time period is defined as “lockdown” for the remainder of this article. We observed the previous temporal pattern of dengue incidences and found that the period of the lockdown coincides between the end of minor (March–May) and the start of major (June–Sept) fluctuations of dengue transmission (Figure 1). Therefore, to understand the heterogeneity of the trend of dengue incidences due to the lockdown, we compared the actual incidence trend of 2020 with three reference trends, namely, mean of incidence of year 2015–2019 (termed as “mean of 2015–2019”), the year 2019 that recorded the highest incidences trend in Malaysia (termed as “year 2019”), and a simulated trend by seasonal autoregressive integrated moving average (SARIMA) models. The SARIMA models mainly served as a reference that presumed the trend of incidences without the interference of city lockdown and population movement control.

To construct the SARIMA models, which are advantageous for modeling the seasonal and time-based dependent configuration of a time series and are commonly applied for epidemiological surveillance [10,11,20], we trained the models by using Malaysia weekly dengue incidences from the 1st week of 2015 to the 52nd week of 2019, and from the dataset, predicted the incidences from the 1st to the 35th week of 2020, including the stages of lockdown in Malaysia (Table 1). We constructed and selected the best SARIMA model (p,d,q) x (P,D,Q) (p is the autoregressive lags, d is the degree of differencing, q is the moving-average lags, P is the seasonal autoregressive lags, D is the seasonal degree of differencing and Q is the seasonal moving-average lags) according to the methods of Box and Jenkins [20]. The best-fitting model was selected based on the lowest values of the normalized Bayesian information criterion (NBIC), the root mean square error (RMSE), and the strongest R-squared (R^2^) value [20]. To validate the prediction ability of the proposed model, the model also applied on the dataset of 2014 dengue incidences (a year that without implementation of lockdown) and predicted the weekly incidences. The performance of the model was evaluated by R^2^ (how well the prediction fits with the actual trend) and RMSE (the differences between predicted and actual incidences).

To analyze the heterogeneity of dengue incidence trend due to the lockdown, we divided the time series of the 1st to the 35th weeks of 2015–2020 into eight stages (two pre-lockdown periods; 8th to 11th weeks, six phases during the lockdown; 12th to 23nd weeks, and one period after the lockdown; 24th to 52nd weeks) according to the announcement by the Malaysia government [21] (Table 1). We compared the dengue incidences for each of the stages by using two-way ANOVA with two independent variables, namely, stages and trends (“year 2020”, “mean of 2015–2019”, “year 2019”, and “SARIMA model”). Because all of the trends follow an open-up parabolic pattern, we further distinguish the pattern by deriving the trend of a particular stage into gradients based on the incidences in three weeks. We compared the magnitude of the gradient of the slope for each of the stages to study the strength of decline, and the stage that consists of a turning point (from negative to positive gradient value), which indicates a resurge of dengue transmission.

### 2.3. Mosquito Occurrences

In order to complete the loop of dengue transmission, we are interested in the component of the vector as well. We start the sampling of Aedes mosquitoes at the 13th week, one week after the Malaysian government announced the movement restriction. The sites were selected from three residential areas, namely, Taman Bukit Jambul (5°20′06.6″ N 100°17′18.7″ E), Taman Permai Indah (5°20′54.4″ N 100°17′52.9″ E) and Taman Jelutong (5°23′18.3″ N 100°18′37.7″ E), which are located within the Northeast Penang Island District and which was the dengue hotspot area before [22]. Due to the restriction of movement order from the Malaysian government [23], the sampling was conducted over a limited coverage of area, with five outdoor (n = 5) and indoor (n = 5) locations from each of the sampling areas, respectively. For high anthropophilic mosquitoes, the human landing catch (HLC) method is the most effective sampling method [24], and one participant was assigned at each of the residential areas for the mosquito sampling. The catch was performed in the early morning from 7:30 A.M. to 10 A.M. with the left arm and both legs exposed without any artificial chemical (e.g., lotion and body shampoo) interference; the mosquitoes were collected by a manual aspirator and transferred to a sealed container. Mosquitoes were killed by freezing, counted, and identified using taxonomy keys. The identification of mosquitoes was based on the distinguishing features—the lyre-shaped markings on *Ae. aegypti* and the white stripe marking on the thorax of *Ae. albopictus*, we also focused on the clypeus-pedicel parts and mesepimeron of *Ae. aegypti* that consisted of distinctive white scales. The counts of *Ae. aegypti* and *Ae. albopictus* from indoor and outdoor locations for the eight stages of the time series during lockdown were obtained and the number of mosquitoes from outdoor was correlated with the weekly reported dengue incidences of Penang, Malaysia by using Spearman’s rank correlation at the significance level of 0.05 (SPSS 17.0, IBM Corp.) Due to the restriction of movement at the beginning of lockdown, indoor sampling started at a stage P3 (16th week), and was therefore not included in the correlation test. All experimental methodologies were approved by the ethical committee of UOW Malaysia KDU Penang University College and in accordance with the guidelines of the ethical committee of UOW Malaysia KDU Penang University College.

## 3. Results

### 3.1. Temporal Analysis of Dengue Incidences during Lockdown

Most of the studies on the temporal analysis of dengue incidences in Malaysia focused on the total cases reported annually, in which the proposed model that was based on yearly basis may not be sensitive and flexible enough to predict the trend and propose necessary dengue control management. To our knowledge, this study is the first to report a SARIMA model with weekly dengue incidences in 2020, including the period of COVID-19 lockdown in Malaysia. To select the best-fitting model, Table 2 shows the comparison of the NBIC, RMSE, MAPE, and R2 of nine SARIMA models, and the best-fitting model for dengue incidences is the SARIMA (1,1,0) (1,1,1) model due to it having the lowest NBIC and RMSE, which infers the lowest errors predicted from the actual trend, and the highest R2, which indicates the strongest correlation of predicted and actual trends. The model also passed the Ljung–Box Q Test (z = 19.782, *p* = 0.180). When the SARIMA model was used to predict the 2014 dengue incidences that consisted of no lockdown, the predicted results showed strong R^2^ values (0.883) and low RMSE (223.66) compared to the actual incidence trends. This support the model in simulating the trend of dengue incidences in 2020 that expects no lockdown.

**Figure 2:**
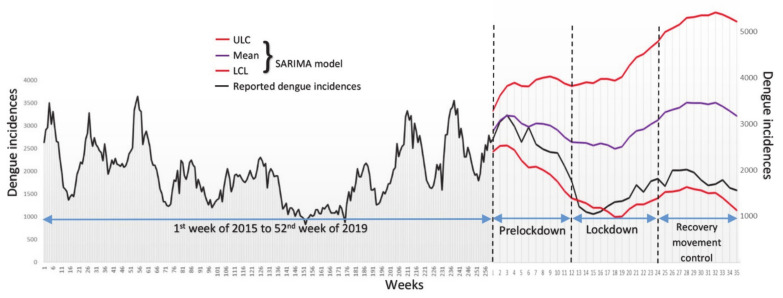
Comparison between actual weekly dengue incidence from the 1st week of 2015 to the 35th week of 2020 with a SARIMA model before and after the duration of partial lockdown. ULC, upper confidence level and LCL, lover confidence level of the SARIMA model.

As seen in Figure 2, when we generated a simulated model on the trend of 13th to the 35th week of 2020, which coincided with the COVID-19 lockdown period in Malaysia, the reported dengue incidence trend of 2020 was significantly diverted and demonstrated a stronger negative steepness compared with the simulated trend. Furthermore, the reported trend of 2020 was lower than the lower confidence level (LCL) of the simulated trend (Figure 2), which implied that the reported dengue incidences of 2020 was significantly lower than the expected trend that presumed no lockdown. Although the underreporting of COVID-19 incidences could be due to other reasons, such as infected people being afraid to visit the hospital due to concern of contraction of COVID-19 from high risk areas, our results nevertheless statistically infer that human movement control greatly impacted dengue transmission in Malaysia.

**Figure 3:**
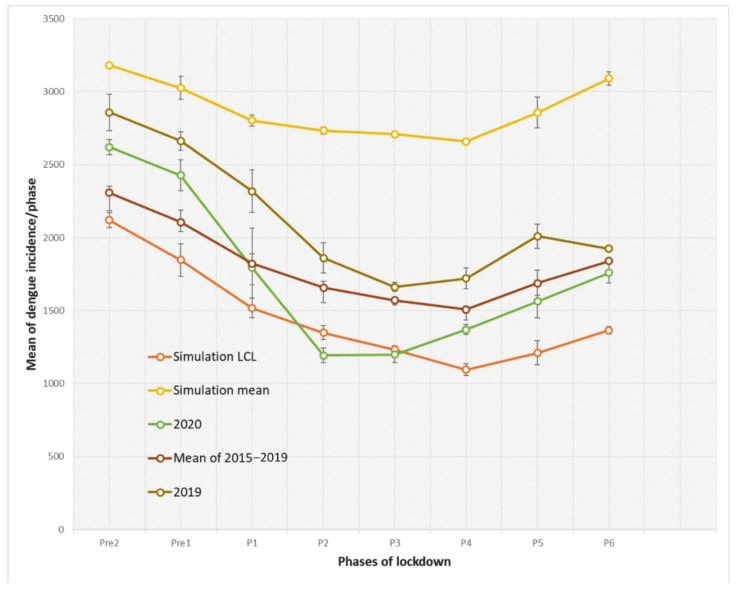
Analysis of variance (ANOVA) of dengue incidences of mean, simulation (SARIMA model) and year 2020 for eight phases of COVID-19 partial lockdown in Malaysia. Pre—Pre-lockdown; P—phase.

From the output of ANOVA, year 2020 had significantly higher dengue incidences at pre-lockdown 1 and 2, and experienced a dramatic decline at the P1 stage (phase 1—12th to 13th weeks of 2020) and eventually consisted of significantly lower incidences at the P2 stage (phase 2—14th to 15th weeks of 2020) (Figure 3) compared to other trends.

To further understand the magnitude of changes of the trends from one stage to another, a gradient of the slope for the particular stage is derived, and Table 3 showed the gradient of the slope for eight stages for the trends of the year 2020, mean 2015–2019, year 2019, and the SARIMA model during the COVID-19 lockdown. As can be seen from the magnitude of the gradient at the P1 stage, when Malaysia imposed phase 1 of the lockdown, the slope declined vividly, which was 319% steeper than in previous years (mean 2015–2019) and 650% steeper than the SARIMA-simulated trend. These provide a strong implication that movement control during the lockdown significantly reduced the reported dengue incidences. Although from the result of ANOVA, at phases 2 to 4 the incidences in 2020 were significantly lower than those in previous years, when we compared the turning point for the gradient changed from negative to positive (which indicates an resurge in dengue incidences), this change in slope occurred in 2020 two stages (4 weeks) earlier than in previous years; specifically, a positive slope was obtained at stage 3 (P3—the 16th to 17th weeks of the year) in 2020 compared to previous years, in which a positive usually was obtained at stage 5 (20th to 23rd weeks of the year). Furthermore, the steepness of the slope of the year 2020 spiked from stage 4 to 5 and had an exponential pattern, suggesting a significant increase in dengue transmission.

### 3.2. Mosquito Occurrences

**Figure 4:**
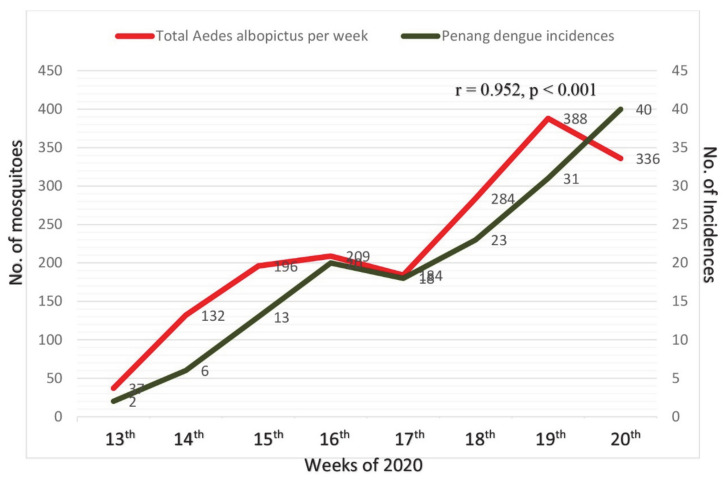
Correlation of total counts of *Aedes albopictus* outdoor sampling from Northeast Penang Island District with number of incidences in Penang.

To study one of the factors that contributes to the spread-out of dengue transmission during the COVID-19 lockdown, we assessed the abundance and distribution of vectors during the breakdown. Figure 4 shows the total numbers of *Ae. albopictus* collected from the outdoor area of the sampling locations during the period of lockdown. *Ae. albopictus* is the predominant species in the outdoor area, while no *Ae. aegypti* was caught. The abundance of *Ae. albopictus* showed slight fluctuation patterns during the lockdown but still demonstrated a strong linear increment throughout the eight stages of lockdown, and showed strong correlation with Penang-reported dengue incidences (Figure 4, r = 0.952, *p* < 0.001). In general, the total number of mosquitoes caught from indoors was significantly lower than outdoors, in which that are a total of 19 *Ae. aegypti* and 37 *Ae. albopictus* from indoors but a total of 1766 per location of *Ae. albopictus* from outdoors (Table 4).

## 4. Discussion

The factors that contribute to dengue transmission are multifaceted, and the spatial variation in the contact rates of the host and vector are probably the most important factors for the dynamics of DENV [25]. With the movement restrictions imposed in Malaysia due to the COVID-19 pandemic, we can investigate the effect of the large-scale movement restrictions of the host on two interrelated variables: dengue transmission and *Aedes* mosquito occurrences. We analyzed the dengue incidence trends by comparing their significant differences among the stages before and during the lockdown to those of the same corresponding periods for previous years and simulations. We first reported that the large-scale human movement restrictions of the COVID-19 lockdown significantly influenced the weekly dengue incidence trend in Malaysia. Our findings provide direct evidence from analysis and extend the studies of Reiner et al. [12] and Falcón-Lezama et al. [7], which demonstrated that people’s movement affected dengue transmission by using a simulation model. The early decline in dengue incidences was also observed in India, with dengue cases dropping by 50% compared to previous years, although without further judgement by statistical analysis. The decline of incidences at the beginning of the lockdown could have occurred for several reasons: (1) fewer hosts available outdoors and therefore less vector–host contact, as *Ae. aegypti* and *Ae. albopictus* are exophilic [26]; (2) the alteration of the environment and relatively fewer artificial breeding sites for the vector due to less solid waste from humans [27]; and (3) the limited movement of infected patients due to the COVID-19 lockdown. Furthermore, some of the infected patients were afraid to visit hospital due to the concern of contraction of COVID-19 from the high risk area [28]. The overwhelming of the healthcare system due to COVID-19 also could be one of the reasons for the underreporting of incidences. As reported in the study of Olive et al. [29], dengue fever patients had delays in diagnosis during the lockdown period, because dengue was considered to be a nonurgent health problem.

Unfortunately, our analysis showed that the dengue incidences rebounded earlier and spiked up at a higher rate than in previous years, indicating that large-scale control of human movement is not sustainable in controlling the spread of dengue. This finding is compatible with the situation in Singapore, which has had the most serious dengue outbreak in seven years [30], and an agent-based simulation model study by Jindal and Rao [31] that showed a significantly higher risk and severity of dengue transmission after the COVID-19 lockdown. The stay-at-home situation makes the host available most of the time in the indoor environment and optimizes the biting activities for endophagic *Ae. Aegypti* to transmit the virus. In contrast to Harrington et al. [32], who argued that people, rather than mosquitoes, rapidly move the virus within and between rural communities and places due to the limitation of the flight range of female *Ae. Aegypti*, our result of *Aedes* mosquitoes revealed that the element of vector dispersal plays a more crucial role in spreading the virus, when the abundance growth of the sampling locations correlated strongly with locally reported dengue incidences. The abundance growth of mosquitoes during the lockdown period could be due to the increase of the potential breeding sites around residential areas, and when people stay at home, more blood meals are available for the egg laying. The high availability of hosts to vectors also explains the early rebound of dengue incidences, especially when the dengue patients had a postponed diagnosis and dengue virus circulation was enhanced within the community; this was also observed in Nacher et al. [33], that when people stayed at home during lockdown, the risk of dengue infection also increased due to the increase of *Ae. aegypti*. With no specific previous entomological data on female adult *Ae. albopictus* in the corresponding period with lockdown, we refer to Rozilawati et al. [14] and Rahim et al. [34], who studied the seasonal abundance of *Ae. albopictus* in Penang by sampling eggs, the Ovitrap index, the container index (CI), the house index (HI) and the Breteau Index (BI). Their results demonstrated that the indexes of *Ae. albopictus* for the corresponding period of stage 2 to 4 of lockdown in this study should be lower, in contrast to our finding that the abundance of *Ae. albopictus* increased steadily from stage 1 to 5. Our result agreed with the study of Reegan et al. [35] in which *Aedes* larval, HI and BI increased significantly during the lockdown period in India. There are several reasons for the increase in *Ae. albopictus* in our study. First, as proposed by the WHO [36], the upsurge of *Aedes* mosquitoes may be due to the southwestern monsoon (end of May to September), which brought a higher frequency of precipitation and higher humidity and temperatures, and therefore, a higher breeding rate for the mosquitoes. Second, a minimum centralized vector control program could be conducted due to the stay-at-home policy. This is relevant to any method that is intended to reduce dengue incidences by reducing, but not eliminating *Aedes* mosquito populations. Before this, researchers [37] have associated the index of the temporal vector with dengue occurrence, and the relationships between *Aedes* mosquito density and DENV transmission indexes for *Ae. aegypti* density are correlated with the prevalence of human dengue infections but are relatively weakly correlated with the incidences, indicating that other factors were involved in determining the incidence pattern. This is supported by the participant during the *Aedes* survey when a fogging activity was observed on 28 May 2020 (22nd weeks of 2020, P5—conditional movement control order), and total *Ae. albopictus* was significantly lower on 29 May 2020, but the mosquitoes caught afterward remained elevated in general.

Furthermore, our findings showed the presence of both *Ae. agypti* and *Ae. albopictus* from an indoor environment but no *Ae. aegypti* from the outdoors, indicating that *Ae. albopictus* is better adapted to a sudden change in the environment, such as the duration of lockdown when most of the hosts shift indoors. Our results are consistent with Nur Aida et al. [38] and Dieng et al. [17], who found that they could increase the invasiveness of *Ae. albopictus* by obtaining a high number of egg and mosquito counts from the indoor environment of Penang Island. Previous studies [13,15,39] from other countries have also reported the aggressive invasive behavior of *Ae. albopictus*, which shared the habitat with other native or existing mosquitoes, including *Ae. aegypti*, which are commonly predominant in indoor environments [40]. Due to the restriction of traveling during the period of lockdown, our results provided limited area coverage, but when considering the scale of the study as a district-level assessment, the results propose a few important discoveries of vector distribution and occurrence during the movement restrictions of lockdown.

## 5. Conclusions

The COVID-19 pandemic is taking hold globally and dengue fever transmission is not on the top of the list of concerns. With a lockdown implemented by Malaysia on 18 March 2020, this study showed that although there was a sharp decrease in dengue incidences in the first two weeks of lockdown, it later rebounded at an earlier time and at a higher rate compared to the corresponding period of previous years and the SARIMA estimation model. Although other factors, such as an overwhelmed healthcare system due to the COVID-19 could also be a reason for the underreporting of incidences, when the host stay at home, it posts a higher possibility for the vector to circulate the virus. Together, without entomological results from a province of Malaysia, our results review the abundance of the *Aedes albopictus*, which increased significantly during the period of lockdown. Therefore, we suggest that *Ae. albopictus* could be the key substitution vector that contributes significantly to dengue virus circulation, and therefore, the vector control direction and strategies should be redesigned.

## Figures and Tables

**Table 1:** Eight Phases of Temporal Analysis of Dengue Transmission with the corresponding start-end date and weeks, and the terms used in Malaysia during COVID-19 lockdown.

Stage	Terms	Start and End Date (Year 2020)	Corresponding Weeks
Pre-2	-	19 February to 3 March	8th to 9th
Pre-1	-	4 March to 17 March	10th to 11th
P1 *	MCO	18 March to 31 March	12th to 13th
P2	MCO	1 April to 14 April	14th to 15th
P3	MCO	15 April to 28 April	16th to 17th
P4	MCO	29 April to 12 May	18th to 19th
P5	CMCO	13 May to 9 June	20th to 23rd
P6	RMCO1	10 June to 31 August	24th to 35th
P7	RMCO2	1 September to 31 December	36th to 52nd

Pre—Prelockdown; P—Phase; MCO—Movement Control Order; CMCO—Conditional Movement Control Order; RMCO—Recover Movement Control Order; * COVID-19 lockdown started on 12th week of 2020.

**Table 2:** Comparison of candidate SARIMA models.

SARIMA Model	Ljung-Box Q Test						
	Statistics (z)	DF	*p*-Value	NBIC	RMSE	MAPE	R^2^
(0,1,0) (0,1,0)	47.293	18	1.92 × 10^−4^	11.615	324.382	13.107	0.743
(0,1,0) (1,1,0)	31.481	17	0.017	11.432	292.239	11.734	0.792
(0,1,0) (1,1,1)	32.411	16	0.009	11.387	282.053	11.311	0.808
(1,1,0) (0,1,0)	20.851	17	0.223	11.526	306.225	12.865	0.772
(1,1,0) (1,1,0)	21.299	16	0.167	11.396	283.304	11.771	0.806
(1,1,0) (1,1,1)	19.782	15	0.180	11.343	272.410	11.344	0.821
(1,1,1) (0,1,0)	20.640	16	0.193	11.554	306.533	12.844	0.773
(1,1,1) (1,1,0)	21.149	15	0.132	11.427	284.056	11.766	0.806
(1,1,1) (1,1,1)	19.746	14	0.138	11.374	273.117	11.333	0.821

NBIC = Normalized Bayesian Information Criterion; RMSE = Root Mean Square Error; MAPE = Mean Absolute Percentage Error; DF: degree of freedom.

**Table 3:** The gradient of the slope for eight stages of the trends during the COVID-19 lockdown.

	Slope *							
	Pre2	Pre1	P1	P2	P3	P4	P5	P6
SARIMA model	−25.60	−134.62	−62.11	−33.75	4.96	−18.36	182.13	74.75
Year 2020	−88	−161	−466	−83	90	57	183.5	44.5
Mean of 2015–2019	−74.1	−140.6	−111.2	−73.2	−19	−11.9	149.5	13.6
Year 2019	−214	−31	−253	−169	−51.5	118	83.5	−221

* The slope is derived from the calculation of the gradient from three weeks’ interval of the particular stage. For example, (incidences at 14th—incidences at 12th)/ two weeks. Pre—Pre-lockdown; P—phase.

**Table 4:** *Aedes* mosquitoes caught from indoor environments in three sampling locations.

Phases *	*Aedes aegypti*	*Aedes albopictus*
P3	4	6
P4	3	5
P5	7	11
P6	5	15

* P-Phase. Due to the restriction of movement at the beginning of lockdown, indoor sampling started at a stage P3 (16th week), and is therefore not included in the correlation test.

## Data Availability

The data presented in this study are available on request from the corresponding author.
